# Lifestyle behaviors and mental health during the coronavirus disease 2019 pandemic among college students: a web-based study

**DOI:** 10.1186/s12889-022-14598-4

**Published:** 2022-11-21

**Authors:** Yi Zhang, Shuman Tao, Yang Qu, Xingyue Mou, Hong Gan, Panfeng Zhou, Zhuoyan Zhu, Xiaoyan Wu, Fangbiao Tao

**Affiliations:** 1 Department of Maternal, Child and Adolescent Health, School of Public Health, Anhui Medical University, No 81 Meishan Road, Hefei, 230032 Anhui China; 2 NHC Key Laboratory of Study on Abnormal Gametes and Reproductive Tract, No 81 Meishan Road, Hefei, 230032 Anhui China; 3 MOE Key Laboratory of Population Health Across Life Cycle, No 81 Meishan Road, Hefei, 230032 Anhui China; 4 Department of Nephrology, The Second Hospital of Anhui Medical University, 678 Furong Road, Hefei, 230601 Anhui China

**Keywords:** Lifestyle health behaviors, Ddepression, Aanxiety, Ccollege students, Ccoronavirus disease 2019

## Abstract

**Background:**

After emerging in China, the novel coronavirus disease 2019 (COVID-19) quickly spread to all parts of the country and became a global public health emergency. The Chinese government immediately took a series of protective and quarantine measures to prevent the spread of the virus, and these measures may have negative effects on behavior and psychological health. This study aimed to examine the associations between factors related to COVID-19 measures and mental health symptoms among Chinese college students in different pandemic areas.

**Methods:**

An online survey was administered to 14,789 college students from February 4 to 12, 2020. After excluding the participants who did not complete the questionnaire, the quality of the questionnaire was checked. Finally, the sample included 11,787 college students from 16 cities and 21 universities in China. The areas included the city of Wuhan (Area 1), the neighboring province of Hubei (Area 2), first-tier cities (Beijing, Shanghai, and Guangzhou [Area 3]), and other provinces (Area 4).

**Results:**

The average age of the participants was 20.51 ± 1.88 years. One-third of the participants were men. In total, 25.9 and 17.8% reported depression and anxiety, respectively. We also explored COVID-19-related factors, such as infection risk, perceived resistance to COVID-19 (or susceptibility to COVID-19 infection), perceived physical symptoms, family or friends, direct or indirect contact with confirmed cases, and having sought psychological counseling, which were significantly associated with anxiety and depression symptoms. Higher screen time, lower physical activity, higher soda and tea beverages (also called sugar sweetened beverages intake), use of alternative medicines or food supplements (including Chinese herbal medicines and vitamins), and decreased meal frequency were all correlated with higher depression and anxiety symptoms (depression: *χ*^2^ = 25.57 and anxiety: *χ*^2^ = 39.42). Coping with COVID-19 partially mediated the associations between some related lifestyle behaviors, anxiety, and depression. The conditional process model analysis results supported our hypotheses that lifestyle health behaviors and coping style were both predictors of anxiety and depression symptoms, and their direct and indirect effects were moderated by sex.

**Conclusions:**

Compared with the city of Wuhan, other epidemic areas had a lower risk of mental health problems. Lifestyle health behaviors and coping styles alleviated mental health symptoms. COVID-19-related social stressors were positively associated with mental health symptoms.

**Supplementary Information:**

The online version contains supplementary material available at 10.1186/s12889-022-14598-4.

## Background

After the first confirmed case in December 2019, coronavirus disease 2019 (COVID-19) rapidly spread to many cities in China and became a public health emergency of international concern (PHEIC) [1, 2]. At the same time, the Chinese government has implemented strict self-quarantine measures and government-mandated quarantines across the country, asking people to stay at home as much as possible and reduce going out to contain the further spread of COVID-19 [3]. In the short term, COVID-19 has become a pandemic that affects physical and mental health and well-being worldwide [4]. During long-term home confinement, lifestyle behavior is likely to change significantly, and psychological health may also be greatly affected [5, 6]. By April 05, 2020, COVID-19 had spread to 210 countries and territories worldwide, and as of May 23, 2020, over 5.1 million confirmed cases and more than 330,000 COVID-19-related deaths had been reported [7].

The above facts suggest an urgent need for improved prevention and treatment for COVID-19-related behaviors that can lead to chronic diseases, particularly in the areas of lifestyle behaviors and mental health symptoms among school-aged children and adults. Previous studies have shown that long-term home confinement leads to changes in daily activities, which include unhealthy diet lifestyles, such as health care product intake and decreased PA and increased ST, these changes in routine activities are aimed at obtaining accurate and up-to-date information on COVID-19 so that better informed decisions can be made to respond to some of the emergencies and improve physical fitness during the pandemic [8, 9]. For example, in Moitra’s study, during the COVID-19 pandemic, only 12% engaged in moderate to vigorous PA and the mean weekday and weekend ST as 442.3 (201.5) minutes/d and 379.9 (178.2) minutes/d, respectively [9]. Another comparative study has reported that time spent on PA decreased (8515.7 ± 10,260.0 vs. 5035.5 ± 5502.0) MET-min·wk.^− 1^ among young adults during the COVID-19 pandemic [10]. These findings demonstrate that the decrease in physical activity level and the increase in screen time are mutually reinforcing and changing to some extent among children and adolescents since the COVID-19 outbreak [10, 11].

COVID-19 outbreak is all of a sudden, the effects of it is also widely, people obtain information through the media, and the spread of the epidemic information through the media (false information and inaccurate reports) overload and may cause significant effects of disasters and emergency communication, to people’s psychological panic, anxiety, anxiety and depression [12, 13]. Furthermore, the change of lifestyle behaviors, such as increasing the intake of sugar sweetened beverages (SSBs) and the use of health care products, sleep rhythm and rhythm than before the diet change, may disrupt the normal rhythm of life, to further increase the mental health problems [12, 14], thus the COVID - 19 during a pandemic mood and behavior have a profound impact on the public [12, 14], causing a profound impact on the public’s mood and behavior during the COVID-19 pandemic. Previous studies have suggested a positive association between the crude infection rate, crude mortality rate, and SSBs. Fruit consumption has been found to negatively affect crude infection and crude mortality rates [15]. Notably, regular sleep habits are crucial for our physical health, so maintaining regular diet rhythmicity and avoiding unnecessarily disrupted rhythms, regardless of sleep or diet, are important for our health [16].

However, few studies have investigated the changes in overall behaviors among young adults during COVID-19, including their effect on college students in different pandemic areas. Moreover, positive coping styles have been found to be associated with decreased negative mental health symptoms [17]. Studies on changes in academic life in schools are scarce; therefore, to the best of our knowledge, the present study is the first to investigate the prevalence of health behaviors among college students during the pandemic. Moreover, we examined lifestyle behaviors and mental health. Specifically, we aimed to evaluate health behaviors and mental health in different pandemic areas. Thus, given that the epidemic may last for weeks to months, maintaining good living habits and developing a suitable physical exercise program and family lifestyle during this period can reduce the negative psychological impact.

## Methods

### Setting and participants

**Fig. 1:**
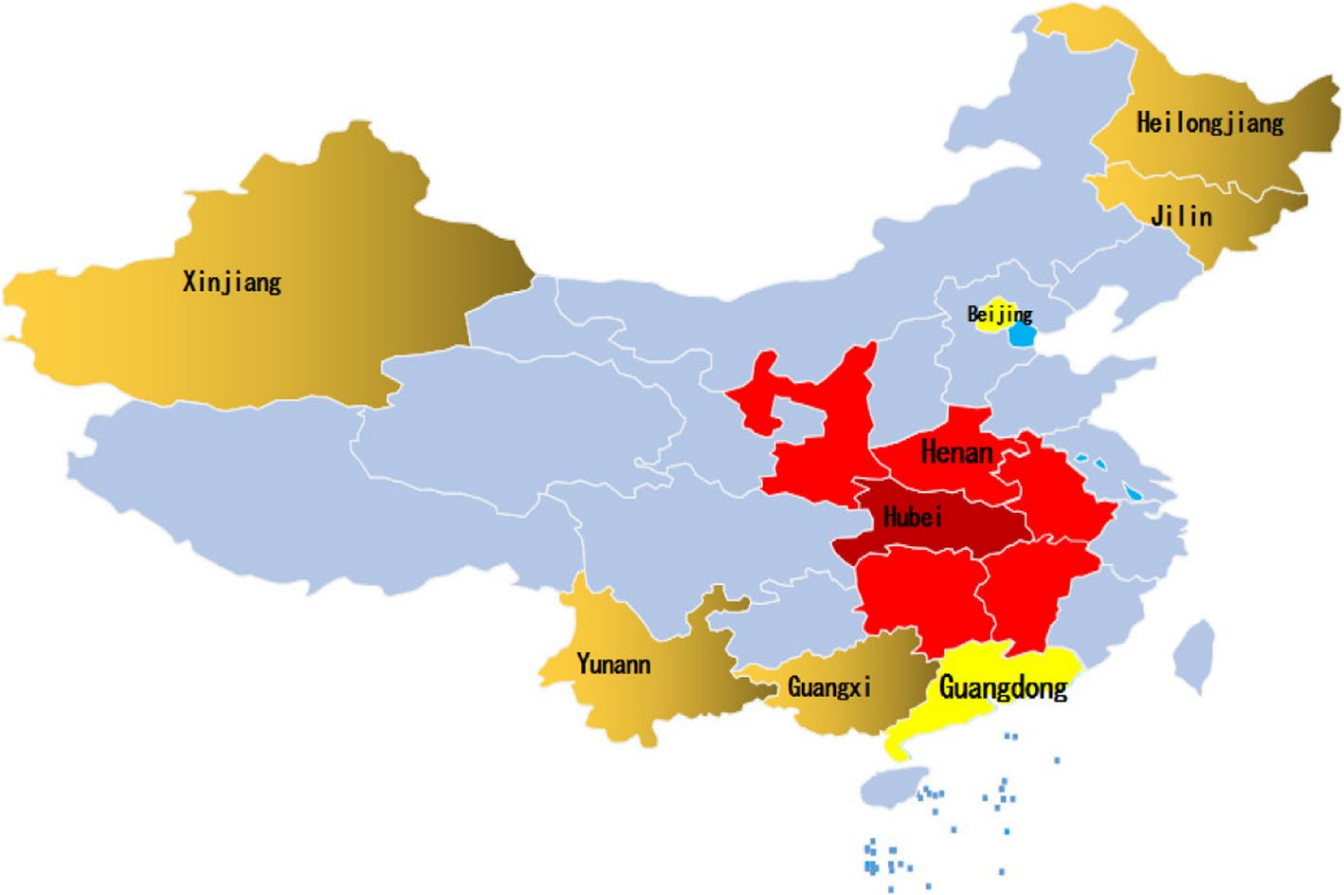
The distribution of sample with the locations of the 16 provinces

A nationwide, cross-sectional online survey was conducted from February 4 to 12, 2020. We considered both the sampling method and the partnership required to carry out the study [18]. We first contacted the Centers for Disease Control and Prevention, and the local Centers for Disease Control and Prevention finally selected schools according to geographical distribution and degree of school cooperation. A two-stage sampling strategy was used. In the first stage, 16 provinces and municipalities were selected according to the geographic location and cooperation intention (see Fig. 1): the city of Wuhan, the neighboring province of Hubei (Henan, Anhui, Jiangxi, Hunan, Chongqing, and Shanxi), first-tier cities (Beijing, Shanghai, and Guangzhou), and other provinces (Jiangsu, Guangxi, Yunnan, Xingjiang, Heilongjiang, and Jilin). Three and four universities were randomly selected in Wuhan and Hubei, respectively, and 15 universities were randomly selected from other provinces or municipalities. In the second stage, one faculty member was randomly selected from each university, and 100–120 students from each grade (in general, 5 years for medical students and 4 years for non-medical students) were invited to participate in the online survey through the Wenjuanxing platform (https://www.wjx.cn/). In total, 14,789 students were selected [18, 19]. Finally, after removing the participants without completed questionnaires, 11,787 participants from 16 cities and 19 colleges in China remained (Supplemental file, Table S1).

#### Inclusion criteria

The inclusion criteria for the participants were as follows: 1) Understood the subject and obtained informed consent from their parents or guardian, 2) college students, 3) had no history of mental illness, and 4) a student attending a chosen school.

#### Exclusion criteria

The exclusion criteria for the participants were as follows: 1) Informed consent was not obtained, 2) not a college student, 3) failure to submit a questionnaire, and 4) congenital or acquired immunodeficiency. A flowchart of the study is shown in Fig. 1.

### Procedure

The participants completed the questionnaires using an online survey platform (“Survey Star [https://www.wjx.cn/]”). The Ethics Committee of Anhui Medical University approved this study. All the respondents provided informed consent. Data collection occurred over several days (February 4 to 12, 2020). Participants could withdraw or decline participation at any time during the study.

### Survey variables

This study examined factors related to the COVID-19 pandemic. The structured questionnaire consisted of questions that covered several aspects: (1) sociodemographic data; (2) COVID-19-related information, such as *i*) I know if someone has had pneumonia in the past 14 days and tourism history of Wuhan or Hubei, *ii*) contact history with COVID-19 patients in the past 14 days, iii) knowledge and concerns about COVID-19, *iv*) precautionary measures against COVID-19 in the past 14 days, and *v*) additional information required for COVID-19; (3) lifestyle behaviors with COVID-19; and (4) mental health symptoms. Sociodemographic data were collected on gender, age, grade, student type, and regional and school areas. Lifestyle behaviors related to COVID-19 were measured in the following aspects: ST, PA, SSBs, meal frequency, healthy food, appetite, and vigor.

The ST of participants was evaluated using one question: “How much time do you spend on ST?” [20]. ST was categorized as > 4 h/d (high), 2–4 h/d (medium) and ≤ 2 h/d (low) [21–23].

Participants’ PA was measured using the question, “On how many days in the last week did you spend at least an hour (60 minutes) on PA?” (That is, the total amount of PA in a day makes your heart beat faster and sometimes your breathing significantly faster)?” The options range from 0 to 7 days [24]; high level PA was defined as at least 3 days per week (PA ≥ 3 days) of [25]. This item has been previously used in recognized student surveys [18, 19]. It has produced valid and reliable responses [26].

SSB intake of participants were evaluated using two questions: “During the last week, how many soda and tea beverages did you drink per day?” The responses were as follows: “none,” “less than one bottle per day,” “one bottle per day,” “two bottles per day,” “three to four bottles per day,” and “more than four bottles per day.”

Meal frequency was evaluated using the following question: “During the last week, did you feel your diet was more regular?” The answers were as follows: “increased,” “unchanged,” and “decreased.”

Alternative medicine or food supplements were evaluated using two questions: “During the last week, did you consume Chinese herbal medicines or vitamins?” The possible answers were “yes” and “no.”

Appetite and vigor changes were evaluated using two questions: Do you feel worse appetite or vigor than before? The answers were: none, sometimes, half of the day, and all the time.

Coping style during COVID-19 was evaluated using the following questions: (1) “Do you usually keep the unhappy memories in mind but never forget them?”; (2) “When you encounter setbacks, do you usually compare them to those of individuals with similar experiences to make sense?”; (3) “In the face of painful events, can it be used to alleviate the pain with positive actions, such as PA?”; and (4) “When you encounter as a sad situation, do you vent anger on others and often lose your temper?” Responses to these questions were based on a four-point scale:1 (“not at all”), 2 (“several days”), 3 (“more than half the day”), and 4 (“nearly every day”). Cronbach α was 0.74.

The mental health status of college students was measured using the Patient Health Questionnaire (PHQ-9) and the Generalized Anxiety Disorder Questionnaire (GAD-7), and the total score was calculated based on previous studies [27, 28]. The total score of GAD-7 was classified as normal (0–4), mild (5–9), moderate (10–14) and severe (15–21) [29]. The PHQ-9 scale was divided into normal (0–4), mild (5–9), moderate (10–14), moderate severe (15–19) and severe (20–27) [28].

### Statistical analysis

Descriptive statistics were calculated for the sociodemographic characteristics, ST, and PA. The chi-square test was used to analyze the prevalence of depression and anxiety in the general data and health behaviors. PHQ-9 and GAD-7 scores were expressed as dichotomous variables. We also built a multiple lifestyle health behavior index (lifestyle health behavior co-occurrence) to measure the overall behaviors during the pandemic. ST was scored as 1 (> 4 h/d), 2 (2-4 h/d), and 3 (≤ 2 h/d). PA was scored as 1 (< 3 days) or 2 (≥3 days). SSB intake, including soda and tea beverages, were scored as 5 (“none”), 4 (“less than one bottle per day”), 3 (“one bottle per day”), 2 (“two to three bottles per day”), or 1 (“more than four bottles per day”). Meal frequency was scored as 3 (increased), 2 (unchanged), or 1 (decreased). The use of alternative medicines or food supplements, including Chinese herbal medicines and vitamins, was scored as 1 (“yes”) or 2 (“no”). Worse appetite and vigor were scored as 1 (“none”), 2 (“sometimes”), 3 (“half of the day”), or 4 (“all the time”). The multiple lifestyle health behavior index was calculated as the sum of the behavioral scores. For coping styles, items 1 and 3 were reverse scoring items, items 2 and 4 were forward scoring items, and the total score of the entries was calculated. Linear regressions were used to calculate the association between sociodemographic characteristics, health behaviors, and mental health, with a significance level of *P* < 0.05. The associations were presented as regression coefficients and 95% confidence intervals (CIs). Statistical analyses were performed using SPSS Windows software version 23.0 (IBM Corporation, Armonk, NY, USA).

## Results

This study covers a sample of 11,787 college students from 21 universities in 16 cities. Their mean age was 20.51 ± 1.88 years. One-third of the students were women. The demographic characteristics of the college students are shown in Table 1. A total of 25.9% (3053/11,787) and 17.8% (2098/11,787) of participants reported symptoms of depression and anxiety, respectively. College students reported that higher ST, lower PA, higher soda beverage and tea beverage (also called SSBs) intake, use of alternative medicines or food supplements (including Chinese herbal medicines and vitamins), and decreased meal frequency were correlated with higher depression and anxiety symptoms. The prevalence of depression and anxiety symptoms were higher in Area 1 than in the other three areas, and the results were statistically different (depression: *χ*^2^ = 25.57 and anxiety: *χ*^2^ = 39.42).

**Table 1:** The prevalence between health behaviors and depression among college students

Demographic variables	Total	Depression				*χ*^*2*^ value
		Mild stress	Moderate stress	Moderate-severe stress	Severe stress	
Gender						
Male	5056(42.9)	709(14.0)	208(4.1)	171(3.4)	43(0.9)	84.35^**^
Female	6731(57.1)	1313(19.5)	361(5.4)	171(2.5)	77(1.1)	
Residential areas						
Rural	5660(48.0)	942(16.6)	286(5.1)	153(2.7)	50(0.9)	6.79
Urban	6127(52.0)	1080(17.6)	283(4.6)	189(3.1)	70(1.1)	
Age (years)						
≤ 19	3860(32.7)	667(17.3)	165(4.3)	85(2.2)	33(0.9)	27.47^**^
20–22	6426(54.5)	1116(17.4)	333(5.2)	224(3.5)	73(1.1)	
≥ 23	1501(12.7)	239(15.9)	71(4.7)	33(2.2)	14(0.9)	
Regional areas						
Area 1	597(5.1)	114(19.1)	38(6.4)	23(3.9)	12(2.0)	25.57^*^
Area 2	2237(19.0)	409(18.3)	97(4.3)	61(2.7)	12(0.5)	
Area 3	2750(23.3)	481(17.5)	134(4.9)	75(2.7)	28(1.0)	
Area 4	6203(52.6)	1018(16.4)	300(4.8)	183(3.0)	68(1.1)	
Students type						
Medical students	5770(49.0)	898(15.6)	255(4.4)	134(2.3)	51(0.9)	46.87^**^
Non-medical students	6017(51.0)	1124(18.7)	314(5.2)	208(3.5)	69(1.1)	
Grade						
Freshmen	2930(24.9)	484(16.5)	134(4.6)	67(2.3)	23(0.8)	22.35
Sophomore	2609(22.1)	453(17.4)	137(5.3)	85(3.3)	32(1.2)	
Junior	2667(22.6)	481(18.0)	123(4.6)	91(3.4)	29(1.1)	
Senior	2314(19.6)	407(17.6)	117(5.1)	71(3.1)	24(1.0)	
Fifth-grade	1267(10.7)	197(15.5)	58(4.6)	28(2.2)	12(0.9)	
ST						
>4 h	5570(47.3)	1126(20.2)	349(6.3)	195(3.5)	71(1.3)	196.15^**^
2–4 h	3706(31.4)	560(15.1)	119(3.2)	57(1.5)	20(0.5)	
≤ 2 h	2511(21.3)	336(13.4)	101(4.0)	90(3.6)	29(1.2)	
PA						
≥ 3 d	3453(29.3)	491(14.2)	99(2.9)	80(2.3)	25(0.7)	95.27^**^
<3 d	8334(70.7)	1531(18.4)	470(5.6)	262(3.1)	95(1.1)	
Soda beverages						
None	8585(72.8)	1413(16.5)	360(4.2)	217(2.5)	84(1.0)	172.88^**^
Less than one bottle	2682(22.9)	505(18.8)	150(5.6)	83(3.1)	24(0.9)	
One bottle	375(3.2)	70(18.7)	49(13.1)	27(7.2)	6(1.6)	
Two to three bottles	109(0.9)	27(24.8)	8(7.3)	12(11.0)	4(3.7)	
More than four bottles	36(0.3)	7(19.4)	2(5.6)	3(8.3)	2(5.6)	
Tea beverages						
None	9374(79.5)	1550(16.5)	418(4.5)	229(2.4)	88(0.9)	157.10^**^
Less than one bottle	1929(16.4)	383(19.9)	98(5.1)	75(3.9)	12(1.1)	
One bottle	383(3.2)	70(18.3)	46(12.0)	26(6.8)	6(1.6)	
Two to three bottles	68(0.6)	14(20.6)	6(8.8)	6(8.8)	2(2.9)	
More than four bottles	33(0.3)	5(15.2)	1(3.0)	6(18.2)	2(6.1)	
Vitamin						
Yes	2302(19.5)	400(17.4)	132(5.7)	90(3.9)	32(1.4)	21.29^**^
No	9485(80.5)	1622(17.1)	437(4.6)	252(2.7)	88(0.9)	
Chinese herbal medicine						
Yes	1312(11.1)	259(19.7)	82(6.3)	86(6.6)	17(1.3)	93.54^**^
No	10,475(88.9)	1763(16.8)	487(4.6)	256(2.4)	103(1.0)	
Worse appetite than before						
None	10,349(87.8)	1400(13.5)	314(3.0)	106(1.0)	24(0.2)	6635.89^**^
Sometimes	1007(8.5)	546(54.2)	166(16.5)	72(7.1)	19(1.9)	
Half of the day	290(2.5)	55(19.0)	68(23.4)	133(45.9)	22(7.6)	
All the time	141(1.2)	21(14.9)	21(14.9)	31(22.0)	55(39)	
Worse vigor than before						
None	9428(80.0)	965(10.2)	181(1.9)	39(0.4)	10(0.1)	8650.21^**^
Sometimes	1752(14.9)	928(53.0)	252(14.4)	93(5.3)	11(0.6)	
Half of the day	414(3.5)	107(25.8)	101(24.4)	156(37.7)	28(6.8)	
All the time	193(1.6)	22(11.4)	35(18.1)	54(28.0)	71(36.8)	
Meal frequency						
Decreased	1157(9.8)	328(28.3)	139(12.0)	111(9.6)	50(4.3)	932.84^**^
Increased	1591(17.5)	336(21.1)	111(7.0)	95(6.0)	30(1.9)	
Unchanged	9039(66.9)	1358(15.0)	319(3.5)	136(1.5)	40(0.4)	

The daily ST > 4 h/d, 2–4 h/d and ≤ 2 h/d of the college students were 47.2, 31.6, and 21.3%, respectively, and PA < 3 d/w and PA ≥ 3 d/w accounted for 70.7 and 29.3%, respectively. Of all the college students, 27.2 and 20.5% consumed soda and tea beverages; 11.1 and 19.5% used Chinese herbal medicines and vitamins, respectively; and 13.7 and 15.6% consumed soda and tea beverages, respectively. Some reported worse sleep and diet rhythmicity than previous conditions. The higher the ST, the higher the depressive symptoms (≥4 h vs 2–4 h mild:20.2% vs. 15.1%, moderate:6.3% vs. 4.2%, moderate-severe:3.5% vs. 1.5%, severe:1.3% vs. 0.5%; *χ*^2^ = 196.15, *P* < 0.001). Lower PA was associated with higher depression symptoms (mild,18.4% vs. 14.2%; moderate,5.6% vs. 2.9%; moderate-severe,3.1% vs. 2.3%; severe,1.1% vs. 0.9%; *χ*^2^ = 95.27, *P* < 0.001). The students’ daily ST > 4 h/d, 2–4 h/d, and ≤ 2 h/d of depression symptoms were 31.3, 20.3, and 22.0%, and PA < 3 d/w and PA ≥ 3 d/w depression symptoms accounted for 28.2 and 20.1%, respectively. Similar results were also observed for anxiety symptoms. Moreover, the prevalence of sleep rhythmicity decreased, remained unchanged, and increased by 27.9, 58.5, and 13.7%, respectively, and the prevalence of diet rhythmicity decreased, remained unchanged, and increased by 17.5, 66.9, and 15.6%, respectively. Other results and gender differences in health behaviors are shown in Tables 1, 2, and 3.

**Table 2:** The prevalence between health behaviors and anxiety among college students

Demographic variables	Total	Anxiety			*χ*^*2*^ value
		mild stress	moderate stress	severe stress	
Gender					
Male	5056(42.9)	509(10.1)	204(4.0)	71(1.4)	45.83^**^
Female	6731(57.1)	956(14.2)	258(3.8)	100(1.5)	
Residential areas					
Rural	5660(48.0)	690(12.2)	223(3.9)	71(1.3)	3.63
Urban	6127(52.0)	775(12.6)	239(3.9)	100(1.6)	
Age (years)					
≤ 19	3860(32.7)	450(11.7)	103(2.7)	45(1.2)	38.80^**^
20–22	6426(54.5)	829(12.9)	298(4.6)	111(1.7)	
≥ 23	1501(12.7)	186(12.4)	61(4.6)	15(1.0)	
Regional areas					
Area 1	597(5.1)	86(14.4)	27(4.5)	23(3.9)	39.42^**^
Area 2	2237(19.0)	278(12.4)	65(2.9)	25(1.1)	
Area 3	2750(23.3)	322(11.7)	120(4.4)	36(1.3)	
Area 4	6203(52.6)	779(12.6)	250(4.0)	87(1.4)	
Students type					
Medical students	5770(49.0)	640(11.1)	191(3.3)	66(1.1)	41.29^**^
Non-medical students	6017(51.0)	825(13.7)	271(4.5)	105(1.7)	
Grade					
Freshmen	2930(24.9)	311(10.6)	73(2.5)	33(1.1)	47.17^**^
Sophomore	2609(22.1)	333(12.8)	123(4.7)	43(1.6)	
Junior	2667(22.6)	370(13.9)	112(4.2)	43(1.6)	
Senior	2314(19.6)	292(12.6)	101(4.4)	40(1.7)	
fifth-grade	1267(10.7)	159(12.5)	53(4.2)	12(0.9)	
ST					
>4 h	5570(47.3)	795(14.3)	241(4.3)	113(2.0)	113.15^**^
2–4 h	3706(31.4)	396(10.7)	84(2.3)	26(0.7)	
≤ 2 h	2511(21.3)	274(10.9)	137(5.5)	32(1.3)	
PA					
≥ 3 d	3453(29.3)	338(9.8)	111(3.2)	33(1.0)	50.60^**^
<3 d	8334(70.7)	1127(13.5)	351(4.2)	138(1.7)	
Soda beverages					
None	8585(72.8)	1049(12.2)	296(3.4)	115(1.3)	115.70^**^
Less than one bottle	2682(22.9)	327(12.2)	112(4.2)	38(1.4)	
One bottle	375(3.2)	59(15.7)	39(10.4)	10(2.7)	
Two to three bottles	109(0.9)	22(20.2)	14(12.8)	5(4.6)	
More than four bottles	36(0.3)	8(22.2)	1(2.8)	3(8.3)	
Tea beverages					
None	9374(79.5)	1107(11.8)	319(3.4)	125(1.3)	94.71^**^
Less than one bottle	1929(16.4)	275(14.3)	96(5.0)	33(1.7)	
One bottle	383(3.2)	63(16.4)	36(9.4)	9(2.3)	
Two to three bottles	68(0.6)	15(22.1)	7(10.3)	2(2.9)	
More than four bottles	33(0.3)	5(15.2)	4(12.1)	2(6.1)	
Vitamin					
Yes	2302(19.5)	327(14.2)	139(6.0)	42(1.8)	49.12^**^
No	9485(80.5)	1138(12.0)	323(3.4)	129(1.4)	
Chinese herbal medicine					
Yes	1312(11.1)	216(16.5)	104(7.9)	25(1.9)	94.89^**^
No	10,475(88.9)	1249(11.9)	358(3.4)	146(1.4)	
Worse appetite than before					
None	10,349(87.8)	851(8.2)	170(1.6)	54(0.5)	6794.77^**^
Sometimes	1007(8.5)	537(53.3)	93(9.2)	31(3.1)	
Half of the day	290(2.5)	63(21.7)	174(60.0)	21(7.2)	
All the time	141(1.2)	14(9.9)	25(17.7)	65(46.1)	
Worse vigor than before					
None	9428(80.0)	504(5.3)	78(0.8)	18(0.2)	8587.62^**^
Sometimes	1752(14.9)	827(47.2)	147(8.4)	26(1.5)	
Half of the day	414(3.5)	99(23.9)	201(48.6)	34(8.2)	
All the time	193(1.6)	35(18.1)	36(18.7)	93(48.2)	
Meal frequency					
Decreased	1157(9.8)	252(21.8)	126(10.9)	54(4.7)	530.62^**^
Increased	1591(13.5)	251(15.8)	105(6.6)	40(2.5)	
Unchanged	9039(76.7)	962(10.6)	231(2.6)	77(0.9)

**Table 3:** The gender difference of health behaviors among college students

Different behavior variables	Gender		
	Male	Female	*P* value
ST			< 0.01
≤ 2 h (low)	1395(27.6)	1116(16.6)	
2–4 h (medium)	1557(30.8)	2149(31.9)	
>4 h (high)	2104(41.6)	3466(51.5)	
PA			< 0.01
≥ 3 d (high)	1700(33.6)	1753(26.0)	
< 3 d (low)	3356(66.4)	4978(74.0)	
Soda beverages			< 0.01
None	3524(69.7)	5061(75.2)	
Less than one bottle	1214(24.0)	1468(21.8)	
One bottle	223(4.4)	152(2.3)	
Two to three bottles	67(1.3)	42(0.6)	
More than four bottles	28(0.6)	8(0.1)	
Tea beverages			< 0.01
None	3524(69.7)	5061(75.2)	
Less than one bottle	1214(24.0)	1468(21.8)	
One bottle	223(4.0)	152(2.3)	
Two to three bottles	67(0.9)	42(0.6)	
More than four bottles	28(0.5)	8(0.1)	
Chinese herbal medicine			< 0.01
No	4421(87.4)	6054(89.9)	
Yes	635(12.6)	677(10.1)	
Vitamin			< 0.01
No	3945(78.0)	5540(82.3)	
Yes	1111(22.0)	1191(17.7)	
Worse vigor than before			< 0.01
None	4095(81.0)	5333(79.2)	
Sometimes	686(13.6)	1066(15.8)	
Half of the day	200(4.0)	214(3.2)	
All the time	75(1.6)	118(1.8)	
Worse appetite than before			< 0.01
None	4429(87.6)	5920(88.0)	
Sometimes	407(8.0)	600(8.9)	
Half of the day	159(3.1)	131(1.9)	
All the time	61(1.2)	80(1.2)	
Meal frequency			< 0.01
Decreased	450(8.9)	707(10.5)	
Increased	749(14.8)	842(12.5)	
Unchanged	3857(76.3)	5182(77.0)	

As shown in Table 3, female students had high ST(> 4 h), low PA(< 3 d), and high frequency of worse appetite than before. Male students consumed soda and tea beverages, used Chinese herbal medicines and vitamins, and had high frequency of worse vigor than before and high frequency of diet.

A comparison of health behaviors among regional areas is shown in Table 4. In Area 1, ST > 4 h was higher than in Areas 2, 3, and 4 (50.4 vs 49.2, 47.7 and 46.0%, respectively). The same results were observed for PA, consumption of soda and tea beverages, decreased meal frequency, and consumption of Chinese herbal medicines and vitamins (all *P* < 0.01).

**Table 4:** The difference regional areas of health behaviors among college students

Different behavior variables	Regional areas				
	Area 1	Area 2	Area 3	Area 4	P value
ST					< 0.01
≤ 2 h (low)	113(18.9)	444(19.8)	591(21.5)	1363(22.0)	
2–4 h (medium)	183(30.7)	692(30.9)	847(30.8)	1984(32.0)	
>4 h (high)	301(50.4)	1101(49.2)	1312(47.7)	2856(46.0)	
PA					< 0.01
≥ 3 d (high)	174(29.1)	682(30.5)	753(27.4)	1844(29.7)	
<3 d (low)	423(70.9)	1555(69.5)	1997(72.6)	4359(70.3)	
Soda beverages					< 0.01
None	416(69.7)	1636(73.1)	2119(77.1)	4414(71.2)	
Less than one bottle	157(26.3)	528(23.6)	543(19.7)	1454(23.4)	
One bottle	17(2.8)	54(2.4)	66(2.4)	238(3.8)	
Two to three bottles	5(0.8)	10(0.5)	15(0.5)	79(1.3)	
More than four bottles	2(0.3)	9(0.4)	7(0.3)	18(0.3)	
Tea beverages					< 0.01
None	469(78.6)	1814(81.1)	2246(81.7)	4845(78.1)	
Less than one bottle	95(15.9)	327(14.6)	409(14.9)	1098(17.7)	
One bottle	21(3.5)	74(3.3)	80(2.9)	208(3.4)	
Two to three bottles	6(1.0)	15(0.7)	11(0.4)	36(0.6)	
More than four bottles	6(1.0)	7(0.3)	4(0.1)	16(0.3)	
Chinese herbal medicine					< 0.01
No	529(88.6)	2017(90.2)	2489(90.5)	5440(87.7)	
Yes	68(11.4)	220(9.8)	261(9.5)	763(12.3)	
Vitamin					< 0.01
No	448(75.0)	1854(82.9)	2261(82.2)	4922(79.3)	
Yes	149(25.0)	383(17.1)	489(17.8)	1281(20.7)	
Worse appetite than before					< 0.01
None	507(84.9)	1978(88.4)	2418(87.9)	5446(87.8)	
Sometimes	63(10.6)	196(8.8)	232(8.4)	516(8.3)	
Half of the day	16(2.7)	46(2.1)	68(2.5)	160(2.6)	
All the time	11(1.8)	17(0.8)	32(1.2)	81(1.3)	
Worse vigor than before					< 0.01
None	455(76.2)	1810(80.9)	2186(79.5)	4977(80.2)	
Sometimes	96(16.1)	329(14.7)	427(15.5)	900(14.5)	
Half of the day	24(4.0)	69(3.1)	98(3.6)	223(3.6)	
All the time	22(3.7)	29(1.3)	39(1.4)	103(1.7)	
Meal frequency					< 0.01
Decreased	73(12.2)	218(9.7)	279(10.1)	587(9.5)	
Increased	68(11.4)	247(11.0)	400(14.5)	876(14.1)	
Unchanged	456(76.4)	1772(79.2)	2071(75.3)	4740(76.4)	

As shown in Table S2, after adjusting for confounding factors, ST > 4 h/d was positively correlated with depression (*OR* = 1.55, 95%*CI*:1.39–1.73) and anxiety symptoms (*OR* = 1.16, 95%*CI*:1.03–1.32) compared with daily ST ≤ 2 h/d. Daily 2–4 h/d of ST was negatively correlated with depression (*OR* = 0.88, 95%*CI*:0.77–0.99) and anxiety symptoms (*OR* = 0.72, 95%*CI*:0.62–0.82) compared with daily ST ≤ 2 h/d. After adjusting for confounding factors, compared with PA < 3 d/w, weekly PA ≥ 3 d/w was positively correlated with depression (*OR* = 1.55, 95%*CI*:1.41–1.71) and anxiety symptoms (*OR* = 1.46, 95%*CI*:1.31–1.63). The students who reported higher consumption of soda and tea beverages, use of Chinese herbal medicines and vitamins, and decreased meal frequency had higher depression and anxiety symptoms.

Table S3 shows the lifestyle health behavior scores stratified by depression and anxiety symptoms. For depression and anxiety, mental health scores increased, and symptom scores decreased, indicating a positive association between mental health symptoms and lifestyle health behaviors.

**Fig. 2:**
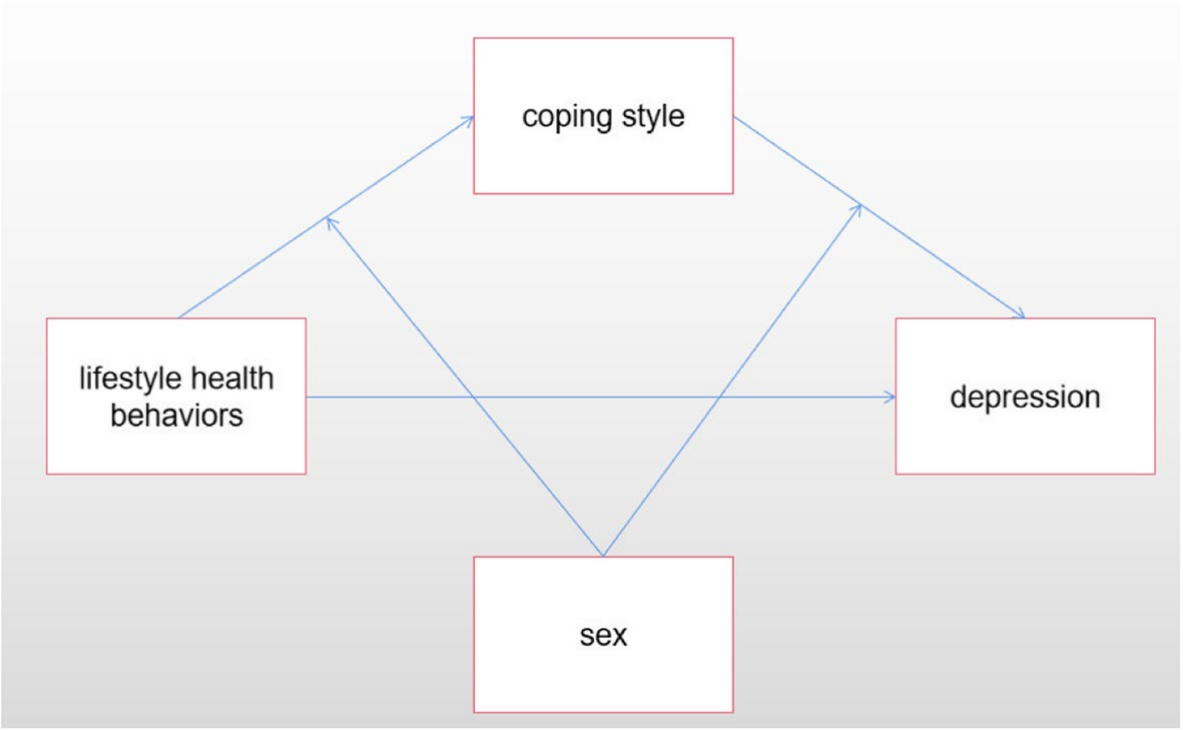
Hypothesis Model

In Tables S4 and S5 and Fig. 2, mediation and moderation analyses were conducted to determine whether the relationships between lifestyle health behaviors and mental health symptoms were (to some extent) mediated by coping responses to COVID-19. This illustrates that there were significant indirect effects (of small magnitude) and significant direct effects (of moderate to large magnitude) on lifestyle health behaviors. Gender moderated only the relationship between lifestyle health behaviors and coping styles.

As shown in Table S6, compared with men, women had higher effects of coping style and better lifestyle on anxiety and depression.

As shown in Fig. S1, COVID-19-related social stressors and mental health were significantly associated with increased levels of anxiety and depressive symptoms: being at higher risk of contracting COVID-19 compared with other people (anxiety: *OR* = 3.58, 95% CI% = 3.0,4.27); depression: *OR* = 2.58, 95% CI% = 2.19,3.05); having perceived lower resistance to COVID-19 (anxiety: *OR* = 3.79, 95% CI% = 3.10,4.64); depression: *OR* = 3.15, 95% CI% = 2.60,3.81), and having family members or friends who had direct or indirect contact with confirmed cases (anxiety: *OR =* 2.20, 95CI% = 1.91,2.53; depression: *OR =* 1.90, 95 CI% = 1.67,2.17). The same results were also shown for students with family or friends living with them who had any travel or residence history in Wuhan, practiced hand washing measures, suspected that they were infected with COVID-19, and sought psychological counseling.

## Discussion

### Principal findings

This large study aim to examine lifestyle behaviors, coping style, COVID-19-related information, and mental health symptoms during the initial epidemic outbreak among college students in China. Our nationwide survey present several main findings. First, approximately half of the ST was more than 4 hours per day, only 21.3% had ST less than 2 hours per day. Simultaneously, some researchers have found the mean ST was 264 min/d in adults and the ST level was also higher [8], which was similar with our results. A systematic review and meta-analysis reported that adults ST changed increased 126.9 ± 42.2 min day^− 1^ [30]. There is related study reported that 1.09% of respondents declared that they did not usually use [31]. These data also showed that the pandemic hit people of different ages, while ST was found to be higher in the results of college students in this study than in a previous study by the research group [25]. Most previous studies examined ST exposure for all age groups, whereas our study focused on college students, who were more susceptible to “infodemic” [31], further contribute to measures that should be taken to reduce sedentary time. Our study also found that more than two thirds (70.7%) of youths had insufficient PA, which was higher than that in adults (nearly 60%) during the pandemic [8]. A total of 55% of students were doing one to three days less aerobic exercise per week during distance education as before [32]. Second, 13.7 and 15.6% of college students had soda and tea beverage intake; 11.1 and 19.5% used Chinese herbal medicines and vitamins, respectively; and 15.6% of college students reported decreased diet rhythmicity. Third, 25.9 and 17.8% of college students reported depression and anxiety symptoms, respectively. Finally, we found that individual and multiple health behavior scores were associated with depression and anxiety symptoms among college students. Moreover, we found that coping styles with COVID-19 partially mediated the associations between some related lifestyle behaviors and anxiety and depression. The conditional process model analysis results supported our hypotheses that lifestyle health behaviors and coping style were both predictors of anxiety and depression symptoms, and their direct and indirect effects were moderated by gender.

The COVID-19 pandemic has posed a severe threat to people’s health and daily life [33]. It also triggers various mental health problems, including anxiety, emotional instability, and depression [12, 13]. In this study, depressive symptoms were higher among college students than in general and COVID-19 surveys [34, 35] and other cross-sectional epidemiological studies [36] conducted among college students by other investigators before the pandemic. Another study reported that a high prevalence of invisible psychological stress caused by ST was positively associated with depressive symptoms as the duration of isolation gradually increased [37]. In a similar previous epidemiological study, adolescents had significantly higher rates of post-traumatic stress disorder (PTSD) symptoms as a result of experiencing isolation [38]. These results and the results of this study all suggest that the psychological impact of the epidemic should not be ignored. Previous experiments on animals have given similar results. Even short periods of isolation (e.g., 24 hours) can also cause anxiety [39], neuropsychiatric disorders [40], and heightened sensitivity to social rewards in adolescent rodents [41]. Animal studies have found that the stress of social isolation in adolescence increases behavioral responses to cocaine in adulthood and alters the responsiveness of brain circuits associated with reward [42]. In particular, it has even more profound effects when rodent adolescent isolation occurs chronically, over one week or longer [43]. Moreover, in our study, we also should paid more attention to lifestyle behaviors and depression and anxiety symptoms among females, non-medical backgrounds, and college students in the high risk area. Females than males were more likely to unhealthy behaviors and depression and anxiety symptoms, which were similar previous studies [8, 12, 18, 44, 45].

Some previous studies have also found that high ST may be positively correlated with high level anxiety and depression; therefore, assessing sedentary based ST could also serve as an important factor in measuring health behaviors in young adults [46, 47]. During COVID-19, our access to information via social media (ST) has increased dramatically, but the quality has not been controlled. Often this information is accompanied by fragmented and unfiltered information [18, 48], which may lead to unnecessary trouble, and when it arrives, bad emotions may also increase [48–50]. Prolonged exposure to ST containing traumatic or threatening content can affect fear conditioning by activating fear circuits in the brain and may produce PTSD symptoms, particularly flashbacks, whereas in this study, panic due to the unknowledge of COVID-19 at the beginning of the outbreak, can lead to changes in emotional behavior caused by prolonged exposure to ST [50]. Interestingly, we also found that college students who maintained intermediate levels of ST were inversely associated with their symptoms of depression and anxiety. One possible reason is that many people are paying attention to COVID-19 related news and information during the pandemic [30]. Further, this suggests that high levels of ST are indeed positively associated with depressive symptoms, but the U-shaped nonlinear association between moderate levels of ST and appropriate information seeking behaviors and depressive and anxiety symptoms may reduce uncertainty and fear induced mental health during the COVID-19 pandemic [51, 52]. Similarly, comparative studies have also demonstrated that, to some extent, appropriate social media approaches, such as engaging in directed communication (i.e., messaging), and ST not exceeding a threshold have been shown to increase happiness [53]. Yet, given the prevalence of information and emotional contagion on online social networks, caution should be exercised when spending excessive time searching for COVID-19 news on ST, especially when screen time exceeds one’s threshold [50]. Additionally, increasing evidence suggests that high PA can bring many physical and mental health benefits [54, 55] and positively affects COVID-19 [56, 57]. Appropriate PA itself can influence the mental health of college students [25, 58, 59]. Similar results have been reported for PA and depressive symptoms during the COVID-19 pandemic [60, 61]. This pattern was also true during the outbreak, suggesting that PA has continued to have health benefits, especially during periods of national isolation, when increased PA could divert excessive ST and further improve people’s physical and mental health.

Our study also explored soda and tea beverage intake. Previous studies have suggested that SSBs can be linked to mental health problems in different age groups [62, 63]. Some studies have demonstrated that a higher intake of SSBs is positively related to COVID-19 infection rates. The same results also demonstrated that intake of fruits had a positive effect on mortality by COVID-19 [15]. In a pandemic such as COVID-19, as students’ lifestyles changed, their thoughts also changed, and SSBs intake caused mental health problems not just by themselves but by a range of behaviors, also known as the theory of planned behavior (TPB) [64]. This included the ST and PA, as previously mentioned. These factors influence each other. Moreover, because of the infodemic, students also buy Chinese herbal medicines (e.g., radix isatidis and Yunnan Baiyao) and vitamins to improve their physical fitness out of unnecessary panic. Sleep is one of the most crucial factors for recovery when someone suffers from diseases or lifestyle events; sleep disturbances can cause anxiety and panic problems during the COVID-19 epidemic [65, 66].

We also explored the students’ meal frequency. We found that students who reported unchanged or increased meal frequency had better mental health, suggesting that in situations like COVID-19, proper timing of diet habits may be necessary to increase unnecessary panic attacks and negative symptoms [67].

To better understand the effect of behaviors on mental health, we adopted a multiple health index, which aimed to explore possible comprehensive influences, including appetite, vigor, diet rhythmicity, SSBs, ST, and PA. It is well known that clustering of health behaviors is also found among students who engage in one lifestyle behavior and are more likely to engage in other lifestyle behaviors [68]. The current study revealed a significant moderating effect of coping style on the development of anxiety and depression symptoms among college students. Coping style was negatively correlated with all indices of distress and positively associated with well-being [69]. More cognitive and prosocial coping behaviors were associated with fewer mental health problems [69]. Moreover, the conditional process model analysis results supported our hypotheses that lifestyle behaviors and coping styles were both predictors of anxiety and depression symptoms, and their direct and indirect effects were moderated by gender.

In this study, nearly 94.7% of students paid close attention to whether they had fever, cough, sneezing, fatigue, etc., and logistic regression showed that perceived physical symptoms (e.g., fever and cough) were significantly associated with higher anxiety and depression symptoms. A previous study has also shown that physical symptoms can significantly impact psychological responses [12, 70]. Physical health perceptions correlate with depression [6]. Family or friends living with students who had direct or indirect contact with someone in the COVID-19 outbreak were also significantly associated with increased anxiety levels. It can be concluded that students were concerned about their family and friends’ health. Additionally, there was no definite treatment at the time, so the related symptoms of anxiety and depression increased [17].

According to the results of the regional division at the beginning of the outbreak, the higher the unhealthy lifestyle behaviors of college students in high-risk areas, the higher the mental health symptoms of college students compared with those in the other three regions. The reason may be that college students in high-risk areas, who live locally, will pay close attention to the development of the epidemic, and therefore, they will have more unhealthy lifestyle behaviors, leading to more mental health symptoms [8, 18]; This also provides a reference for the subsequent research, we should always pay attention to the psychological status of college students in the outbreak areas. The occurrence of mental health was further reduced by combining it with an increase in healthy lifestyle behaviors.

### Strengths and limitations

Our study has some limitations. First, this was a cross-sectional study, we could not establish a causal relationship between lifestyle behaviors and mental health outcomes. Second, lifestyle behaviors and mental health were self-reported, and because of the special epidemic situation, we could not meet with the students face to face; therefore, there might have been recall bias. Third, the mechanisms underlying the associations observed in the study have not been directly assessed. Finally, we have not obtain information on mental health conditions or lifestyle behaviors before the outbreak. We couldn’t compare screen time with changes in physical activity and sugary drinks in our own study.

Despite these limitations, our study deserves further attention. Firstly, we have a large number of samples, which are representative to a certain extent. Our study includes different endemic areas, which can also be used to compare regional differences. Therefore schools and governments in different places can respond quickly. The second is to grasp the epidemic problems in time to provide some reference for the psychological research of domestic college students, especially the psychological factors research and intervention. Our study is a continuous follow-up study, and a follow-up investigation will be conducted every 6 months for up to 2 year, thus enabling further clarification of the relationship. Third, during the quarantine period, on the one hand, we paid close attention to the epidemic information concerned by college students through research and investigation, which was helpful for further targeted understanding of the adverse effects caused by the current situation of the epidemic. On the other hand, as a result of quarantine, people’s lives and movements have been restricted to different degrees, especially those who have had direct and indirect contact with confirmed cases, and may have different degrees of psychological impact during home quarantine. Therefore, this study takes this as a starting point to conduct a nationwide survey on college students’ mental health [18].

## Conclusions

Our study revealed that adopting healthy lifestyle behaviors and timely realization of related COVID-19-influenced factors were negatively associated with mental health. Compared to cities at high risk during the pandemic, the risk of mental health problems in other affected areas is relatively lower and still higher than before. The relevant government departments should also pay attention to the health education of college students with non-medical background and improve the awareness of COVID-19 among college students. These institutions should find methods of making related activities, such as decreasing ST and unhealthy diet patterns and increasing PA and rhythmic rhythm of life more attractive to college students, and increase the knowledge of popular science among this group during the epidemic. Thus, a better understanding of the relationship between healthy lifestyle behaviors and mental health of college students during the epidemic will help school leaders and the Ministry of Education to identify and adopt effective policies and interventions for college students during the outbreak stage, as well as timely control public opinion [11].

## Supplementary Information

**Additional file 1: Table S1.** List of all the participants. **Table S2.** The association between health behaviors and mental health among college students. **Table S3.** Mental health scores stratified by depression, anxiety symptoms. **Table S4.** The mediation effect of coping style on the relationship between lifestyle health behaviors and mental health symptoms in college students. **Table S5.** Model characteristics for the conditional process analysis. **Table S6.** Bootstrapped conditional direct and indirect effects. **Fig. S1.** The correlation between COVID-19 related related social stressors and mental health.

## Data Availability

The datasets generated and/or analysed during the current study are not publicly available but are available from the corresponding author on reasonable request.

## Abbreviations

ST: Screen Time
PA: Physical Activity
SSBs: Sugar sweetened beverages
COVID-19: The 2019 coronavirus disease
PHQ: The patient health questionnaire
GAD: The generalized anxiety disorder questionnaire
PHEIC: Public Health Emergency of International Concern
CIs: Confidence intervals
