# Correction to: Lifestyle behaviors and mental health during the coronavirus disease 2019 pandemic among college students: a web-based study

**DOI:** 10.1186/s12889-023-15591-1

**Published:** 2023-06-22

**Authors:** Yi Zhang, Shuman Tao, Yang Qu, Xingyue Mou, Hong Gan, Panfeng Zhou, Zhuoyan Zhu, Xiaoyan Wu, Fangbiao Tao

**Affiliations:** 1 Department of Maternal, Child and Adolescent Health, School of Public Health, Anhui Medical University, No 81 Meishan Road, Hefei, 230032 Anhui China; 2 NHC Key Laboratory of Study on Abnormal Gametes and Reproductive Tract, No 81 Meishan Road, Hefei, 230032 Anhui China; 3 MOE Key Laboratory of Population Health Across Life Cycle, No 81 Meishan Road, Hefei, 230032 Anhui China; 4 Department of Nephrology, The Second Hospital of Anhui Medical University, 678 Furong Road, Hefei, 230601 Anhui China

**BMC Public Health (2022) 22:2140**

10.1186/s12889-022-14598-4

The original publication of this article was missing the following funding acknowledgement:

- Collaborative Innovation Project of Anhui Provincial Universities(GXXT-2020-068).

The original article has been updated.

