# Supplementary material for: Lifestyle behaviors and mental health during the coronavirus disease 2019 pandemic among college students: a web-based study
Source: BMC Public Health. 2022 Nov 21;22:2140. doi: 10.1186/s12889-022-14598-4 (PMC9682808; doi:10.1186/s12889-022-14598-4)
Supplement: Supplementary file 1 — Additional file 1: Table S1. List of all the participants. Table S2. The association between health behaviors and mental health among college students. Table S3. Mental health scores stratified by depression, anxiety symptoms. Table S4. The mediation effect of coping style on the relationship between lifestyle health behaviors and mental health symptoms in college students. Table S5. Model characteristics for the conditional process analysis. Table S6. Bootstrapped conditional direct and indirect effects. Fig. S1. The correlation between COVID-19 related related social stressors and mental health. [file 12889_2022_14598_MOESM1_ESM.doc]

**Supplemental file 1**

**Table S1 List of all the participants**

| **Location** | **Number of Universities** | **Faculties** | **Grade** | **Total** | **Valid sample** |
| --- | --- | --- | --- | --- | --- |
| Anhui | 1 | Medical×1 | Year 1-5 | 500 | 500 [100 per grade] |
| Heilongjiang | 1 | Non-medical×1 | Year 1-4 | 400 | 400 [100 per grade] |
| Guangdong | 1 | Medical×1 | Year 1-5 | 500 | 500 [100 per grade] |
| Guangxi | 1 | Non-medical×1 | Year 1-4 | 400 | 400 [100 per grade] |
| Jiangsu | 1 | Non-medical×1 | Year 1-4 | 400 | 400 [100 per grade] |
| Wuhan, Hubei [1] | 1 | Medical×1 | Year 1 | 135 | 111 |
|  |  | Medical×1 | Year 2 | 125 | 88 |
|  |  | Medical×1 | Year 3 | 138 | 66 |
|  |  | Medical×1 | Year 4 | 138 | 105 |
|  |  | Medical×1 | Year 5 | 146 | 146 |
| Jilin | 1 | Medical×1 | Year 1-5 | 500 | 500 [100 per grade] |
| Yunan | 1 | Medical×1 | Year 1-5 | 500 | 500 [100 per grade] |
| Shanghai | 1 | Non-medical×1 | Year 1-4 | 400 | 400 [100 per grade] |
| Jiangxi | 1 | Non-medical×1 | Year 1-4 | 400 | 400 [100 per grade] |
| Beijing | 1 | Medical×1 | Year 1 | 74 | 74 |
|  |  | Medical×1 | Year 2 | 76 | 69 |
|  |  | Medical×1 | Year 3 | 86 | 86 |
|  |  | Medical×1 | Year 4 | 79 | 79 |
|  |  | Medical×2 | Year 5 | 230 | 192 |
| Wuhan, Hubei [2] | 1 | Medical×1 / Non-medical×1 | Year 1 | 235 | 163 [Medical:76] |
|  |  | Medical×1 / Non-medical×1 | Year 2 | 330 | 131 [Medical:77] |
|  |  | Medical×1 / Non-medical×1 | Year 3 | 255 | 104 [Medical:40] |
|  |  | Medical×1 / Non-medical×1 | Year 4 | 327 | 102 [Medical:43] |
|  |  | Medical×1 / Non-medical×1 | Year 5 | 134 | 59 [Medical:58] |
| Wuhan, Hubei [3] | 1 | Medical×1 / Non-medical×8 | Year 1 | 1341 | 1044 [Medical:37] |
|  |  | Medical×1 / Non-medical×8 | Year 2 | 1400 | 859 [Medical:79] |
|  |  | Medical×1 / Non-medical×8 | Year 3 | 1378 | 938 [Medical:88] |
|  |  | Medical×1 / Non-medical×8 | Year 4 | 1281 | 568 [Medical:104] |
|  |  | Non-medical×1 | Year 5 | 0 | 3 |
| Xi’an | 1 | Non-medical×1 | Year 1 | 100 | 100 |
|  |  | Medical×1 | Year 2 | 100 | 100 |
|  |  | Non-medical×1 | Year 3 | 100 | 100 |
|  |  | Medical×1 / Non-medical×1 | Year 4 | 100 | 100 [Medical:52] |
|  |  | Medical×1 | Year 5 | 100 | 100 |
| Xinjiang | 1 | Medical×1 | Year 1-5 | 500 | 500 [100 per grade] |
| Henan | 1 | Medical×1 | Year 1-5 | 500 | 500 [100 per grade] |
| Hunan | 1 | Medical×1 | Year 1 | 146 | 135 |
|  |  |  | Year 2 | 82 | 57 |
|  |  |  | Year 3 | 73 | 70 |
|  |  |  | Year 4 | 79 | 70 |
|  |  |  | Year 5 | 78 | 68 |
| Wuhan, Hubei [4] | 1 | Non-medical×1 | Year 1-4 | 400 | 400 [100 per grade] |
| Chongqing | 1 | Medical×1 | Year 1 | 118 | 103 |
|  |  |  | Year 2 | 105 | 105 |
|  |  |  | Year 3 | 103 | 103 |
|  |  |  | Year 4 | 94 | 90 |
|  |  |  | Year 5 | 103 | 99 |
| **Total** | **19** |  |  | **14789** | **11787** |

| Table S2 The association between health behaviors and mental health among college students | | |
| --- | --- | --- |
| Different behavior variables | Anxiety and depression | |
|  | Anxiety *OR*(95 % *CI*) | Depression *OR*(95 % *CI*) |
| ST |  |  |
| ≤2 h (low) | 1.00 | 1.00 |
| 2-4 h (medium) | 0.72(0.62-0.82)^**^ | 0.88(0.77-0.99)^*^ |
| ＞4 h (high) | 1.16(1.03-1.32)^*^ | 1.55(1.39-1.73)^**^ |
| PA |  |  |
| ≥3 d (high) | 1.00 | 1.00 |
| ＜3 d (low) | 1.46(1.31-1.63)^**^ | 1.55(1.41-1.71)^**^ |
| Soda beverages |  |  |
| None | 1.00 | 1.00 |
| Less than one bottle | 1.09(0.97,1.22) | 1.28(1.16-1.41)^**^ |
| One bottle | 2.16(1.71,2.73)^**^ | 2.36(1.90-2.93)^**^ |
| Two to three bottles | 3.21(2.16,4.77)^**^ | 3.07(2.09-4.50)^**^ |
| More than four bottles | 2.71(1.35,5.48)^**^ | 2.25(1.14-4.429)^*^ |
| Tea beverages |  |  |
| None | 1.00 | 1.00 |
| Less than one bottle | 1.38(1.22,1.56)^**^ | 1.36(1.22,1.52)^**^ |
| One bottle | 2.03(1.61,2.56)^**^ | 2.02(1.63,2.50)^**^ |
| Two to three bottles | 3.10(1.87,5.14)^**^ | 2.41(1.48,3.94)^**^ |
| More than four bottles | 2.92(1.41,6.07)^**^ | 2.57(1.28,5.15)^**^ |
| Chinese herbal medicine |  |  |
| No | 1.00 | 1.00 |
| Yes | 1.80(1.58,2.06)^**^ | 1.58(1.40,1.79)^**^ |
| Vitamin |  |  |
| No | 1.00 | 1.00 |
| Yes | 1.41(1.26,1.58)^**^ | 1.18(1.06,0.31)^**^ |
| Worse appetite than before |  |  |
| None | 1.00 | 1.00 |
| Sometimes | 4.41(3.69,5.26)^**^ | 5.01(4.13,6.08)^**^ |
| Half of the day | 11.51(7.23,17.21)^**^ | 14.60(7.65,27.83)^**^ |
| All the time | 2.05(1.22,3.47)^**^ | 6.05(2.94,12.46)^**^ |
| Worse vigor than before |  |  |
| None | 1.00 | 1.00 |
| Sometimes | 10.79(9.35,12.45)^**^ | 10.87(9.50,12.44)^**^ |
| Half of the day | 24.43(18.24,32.72)^**^ | 51.76(32.93,81.35)^**^ |
| All the time | 55.75(34.90,89.06)^**^ | 55.97(29.43,106.47)^**^ |
| Frequency of diet |  |  |
| Decreased | 1.00 | 1.00 |
| Increased | 0.27(0.24,0.31)^**^ | 0.33(0.30,0.36)^**^ |
| Unchanged | 0.57(0.48,0.67)^**^ | 0.36(0.32,0.42)^**^ |
| Adjusted for age, grade, gender, student types, regional areas, residential areas. | | |

| Table S3 Mental health scores stratified by depression, anxiety symptoms. | | | | |
| --- | --- | --- | --- | --- |
| Mental health (Range of Scores) | N(%) | Health behaviors score mean (SD) | *R^2^* | B(95% CI) |
| Depression |  |  | 0.257 | -1.22(-1.26,-1.18)^**^ |
| None | 8734(74.1) | 24.93(1.44) |  |  |
| Mild | 2022(17.2) | 23.67(1.93) |  |  |
| Moderate | 569(4.8) | 22.65(2.31) |  |  |
| Moderate-severe | 342(2.9) | 21.18(2.60) |  |  |
| Severe | 120(1.0) | 19.98(2.65) |  |  |
| Anxiety |  |  | 0.216 | -1.50(-1.55,-1.45)^**^ |
| None | 9689(82.2) | 24.81(1.55) |  |  |
| Mild | 1465(12.4) | 23.33(2.04) |  |  |
| Moderate | 462(3.9) | 21.71(2.62) |  |  |
| Severe | 171(1.5) | 20.43(2.80) |  |  |

| Table S4 The mediation effect of coping style on the relationship between lifestyle health behaviors and mental health symptoms in college students | | | | |
| --- | --- | --- | --- | --- |
|  | Anxiety | | Depression | |
|  |  | *P* value |  | *P* value |
| Lifestyle behaviors→coping style | 0.1017 | <0.001 | 0.1017 | <0.001 |
| Lifestyle behaviors→mental health | -0.9142 | <0.001 | -1.2344 | <0.001 |
| Coping style→mental health | -0.2734 | <0.001 | -0.3546 | <0.001 |
| Direct lifestyle behaviors effect | -0.9142 | <0.001 | -1.2344 | <0.001 |
|  |  | 95%CI |  | 95%CI |
| Indirect coping style effect | -0.0278 | -0.0344,-0.0221 | -0.0360 | -0.0442,-0.0293 |
| Mediate variables: coping style, independent variables: lifestyle health behaviors, dependent variables: mental health symptoms. The model was controlled for age, grade, gender, student types, regional areas, residential areas. | | | | |

| Table S5 Model characteristics for the conditional process analysis. | | | | |
| --- | --- | --- | --- | --- |
|  | Anxiety | | Depression | |
|  | B | *P* value |  | *P* value |
| Lifestyle behaviors | -0.1695 | <0.001 | -0.2136 | <0.001 |
| Coping style | -0.9365 | <0.001 | -1.1182 | <0.001 |
| Sex | 0.8251 | 0.3149 | 3.4961 | <0.001 |
| Lifestyle behaviors *** gender | -0.0639 | 0.1224 | -0.0849 | 0.0943 |
| Gender * Lifestyle behaviors | 0.0149 | 0.6259 | -0.0767 | 0.0414 |
| R^2^ | 0.2620 | | 0.2983 | |
| F | 418.13 | | 500.55 | |
| Mediate variables: coping style, moderated variables: sex, independent variables: lifestyle health behaviors, dependent variables: mental health symptoms. The model was controlled for age, grade, student types, regional areas, residential areas. | | | | |

| Table S6 Bootstrapped conditional direct and indirect effects. | | | | | | | | |
| --- | --- | --- | --- | --- | --- | --- | --- | --- |
|  |  | | Anxiety | | | Depression | | |
| Direct effect |  |  | Effect | SE | (LL,UL) | Effect | SE | (LL,UL) |
|  | Predictor | Lifestyle health behaviors |  |  |  |  |  |  |
|  | Moderator | Male | -0.9215 | 0.0219 | -0.9645,-0.8786 | -1.1949 | 0.0269 | -1.2476,-1.1423 |
|  |  | Female | -0.9066 | 0.0215 | -0.9488,-0.8644 | -1.2716 | 0.0264 | -1.3234,-1.2198 |
| Indirect effect |  |  | Effect | SE | (LL,UL) | Effect | SE | (LL,UL) |
|  | Predictor | Coping style |  |  |  |  |  |  |
|  | Moderator | Male | -0.0156 | 0.0032 | -0.0227,-0.0102 | -0.0199 | 0.004 | -0.0281,-0.0126 |
|  |  | Female | -0.0401 | 0.0053 | -0.0518,-0.0309 | -0.0518 | 0.0064 | -0.0663,-0.0410 |
| Mediate variables: coping style, moderated variables: sex, independent variables: lifestyle health behaviors, dependent variables: mental health symptoms. The model was controlled for age, grade, student types, regional areas, residential areas. | | | | | | | | |


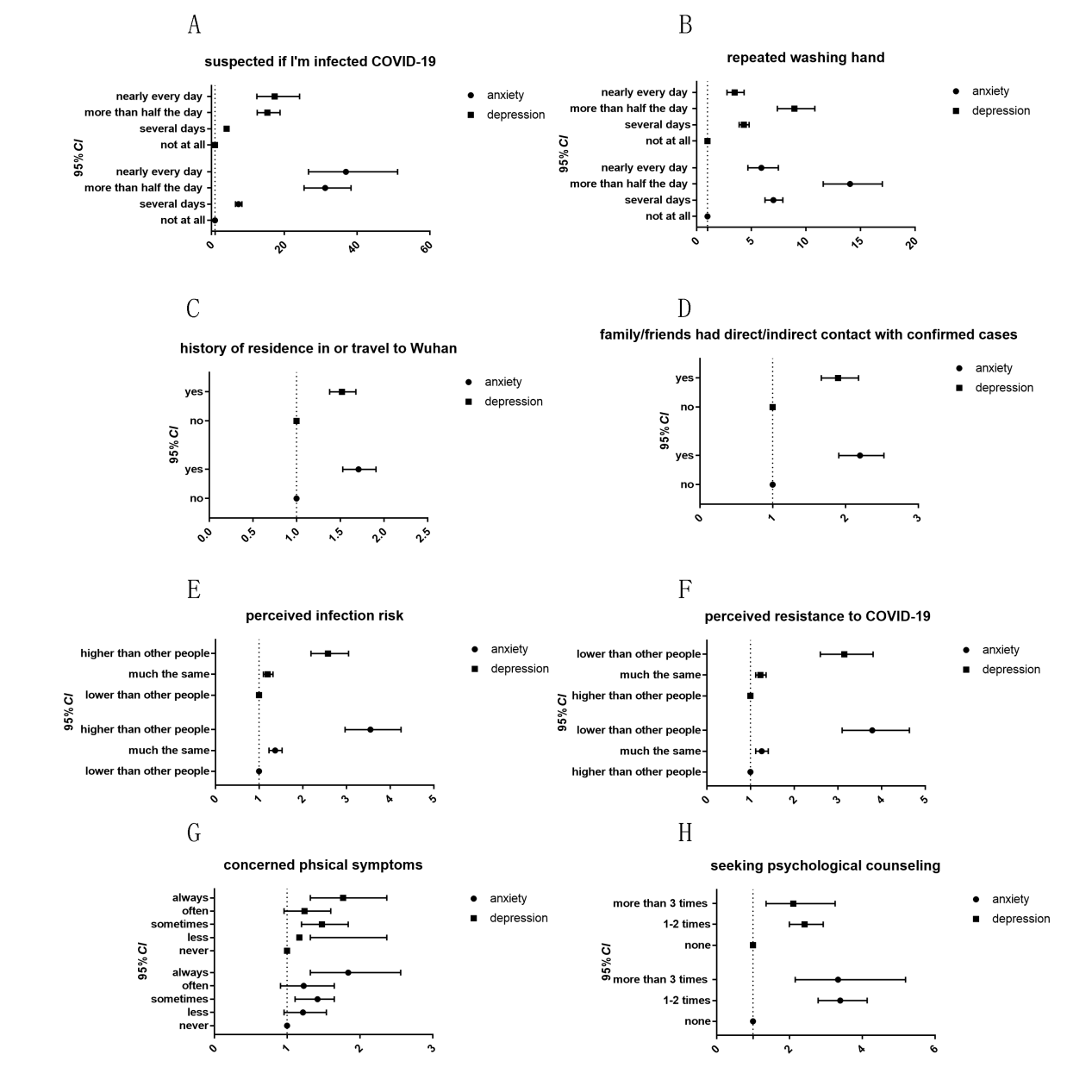


**Fig S1 The correlation between COVID-19 related related social stressors and mental health**
